# Molecular Alterations of Circulating Cell-Free DNA in the Pathological Progression of Hepatocellular Carcinoma

**DOI:** 10.1155/2021/3637436

**Published:** 2021-12-03

**Authors:** Wenbo Guo, Jilin Lu, Linlin Yan, Debin Sun, Longlong Gong, Wei Shi

**Affiliations:** ^1^Department of Interventional Radiology, The First Affiliated Hospital of Sun Yat-Sen University, Guangzhou, China; ^2^Department of General Surgery, Huashan Hospital, Fudan University, Shanghai, China; ^3^Department of Infectious Disease, Center for Liver Disease, Peking University First Hospital, Beijing, China; ^4^Genecast Biotechnology Co., Ltd., Xidong Chuangrong Building, Wuxi, Jiangsu, China

## Abstract

**Background:**

Hepatocellular carcinoma (HCC) is one of the most malignant cancers. Early diagnosis of HCC is important to reduce the mortality rate. The aim of this study is to explore the plasma cell-free DNA (cfDNA) mutation profile in the pathological progression of HCC and to investigate the significance of plasma cfDNA mutations in the early diagnosis of HCC.

**Methods:**

Thirty-seven patients with chronic hepatitis B (CHB), eight with liver cirrhosis (LC), and eleven with HCC were enrolled in this cohort. Plasma cfDNA and white blood cell DNA were isolated, and plasma cfDNA mutation profiles were detected using a targeted gene panel.

**Results:**

The sequencing results of plasma cfDNA showed that HCC-related gene mutations were present in patients with CHB and LC. The mutation burden of HCC-related genes increased from CHB and LC to HCC. In patients with HCC, the average mutation burden of NRAS (10.1%), TP53 (7.4%), PTEN (4.2%), and APOB (2.6%) was the highest. The average mutation burden of PTEN, APOB, FRAS1, KDM6A, DDR2, TTK, NRAS, TP53, PTPRB, MPL, FCRL1, HN1, and SFN gradually increased from CHB and LC to HCC. The mutation burden of 18 HCC-related genes had an area under the receiver operating characteristics of 0.92 for the diagnosis of HCC.

**Conclusions:**

The mutation burden of HCC-related genes increased from CHB and LC to HCC. An optimal combination of cfDNA mutations in the gene panel for diagnosing HCC in patients with CHB and LC was selected. Our study indicates that somatic mutations in plasma cfDNA may serve as potential biomarkers for early HCC diagnosis.

## 1. Introduction

Hepatocellular carcinoma (HCC) is the sixth most common cancer worldwide and the third most deadly cancer [1]. Viral infection, especially hepatitis B virus, increases the risk of liver cirrhosis (LC) and HCC [2–4], with approximately 60–65% of HCC cases caused by hepatitis B virus infection [5]. Reports show that 1.3% of patients with LC develop HCC each year [6], underscoring the necessity of surveillance for cirrhotic patients. Generally, the high mortality rate of HCC is due to the low rate of early diagnosis, lack of clinically effective systemic treatment, high intratumoral heterogeneity, and high relapse rates.

Liver biopsy is the gold standard for the diagnosis of liver diseases despite its invasiveness. However, it is unlikely that the biopsy can reflect the entire liver condition owing to the tissue heterogeneity. Current methods such as alpha-ferroprotein blood tests and imaging modalities lack sensitivity and specificity, which limits their use in early diagnosis. The use of liquid biopsies has been developed and validated over the past few years, making the diagnosis of HCC and liver fibrosis possible [7–9].

Circulating cell-free DNA (cfDNA) detected in the blood of cancer patients carries tumor genomic alterations and has been widely investigated in various cancers as a biomarker for diagnosing, predicting, and monitoring response to therapy and monitoring tumor burden and relapse due to its comprehensive capture of somatic mutations [10–13]. Several studies have shown that the level of cfDNA is markedly elevated in patients with HCC and can be detected early in tumorigenesis [14–16]. Therefore, somatic mutations in cfDNA may provide necessary information to distinguish between benign and malignant tumors. To date, there have been only a few studies that utilized cfDNA to track the development of chronic hepatitis B (CHB), LC, and HCC [17–19]; hence, further research is required to better understand the genetic mutational progression in plasma cfDNA of patients with CHB, LC, and HCC. The present study was conducted to profile the plasma cfDNA mutations in the pathological progression from CHB and LC to HCC and to investigate the significance of plasma cfDNA mutations in the early screening for HCC.

## 2. Methods and Materials

### 2.1. Patients and Sample Collection

Thirty-seven patients with CHB, eight with LC, and eleven with HCC were enrolled from the First Affiliated Hospital of Sun Yat-sen University in this cohort. The collected samples were sequenced by Genecast Biotechnology Co., Ltd. (Beijing, China). This study was approved by the Clinical Research and Experimental Animal Ethics Committee of the First Affiliated Hospital of Sun Yat-sen University (No. 2019239) and was performed in accordance with the principles of the Declaration of Helsinki. All participants provided written informed consent.

### 2.2. Plasma DNA Extraction

Blood samples (10 mL) were collected from all participants, and the plasma was separated by centrifugation. The MagMAX^TM^ Cell-Free DNA Isolation Kit (Thermo Fisher Scientific, Waltham, MA, USA) was used to isolate plasma cfDNA according to the manufacturer's instructions. The TIANamp Blood DNA Kit (TIANGEN, Beijing, China) was used to extract white blood cell DNA. The extracted DNA was quantified using a Qubit 4.0 Fluorometer (Thermo Fisher Scientific, Waltham, MA, USA).

### 2.3. Library Construction and cfDNA Sequencing

Genomic DNA extracted from white blood cells was fragmented into 200-bp DNA pieces with a M220 Focused-Ultrasonicator (Covaris, Woburn, MA) according to the manufacturer's protocol. Both the cfDNA library and the genomic DNA library were constructed using a KAPA Hyper Preparation Kit (Kapa Biosystems, Wilmington, MA). A 2100 bioanalyzer with a DNA 1000 kit (Agilent, Santa Clara, CA) was used to determine the fragment length. DNA was hybridized to a designed 543-gene panel (Genecast, Wuxi, China) that included major tumor-related genes, covering 1.7 Mb of the genome. The sequencing libraries were quantified using a Qubit dsDNA HS Assay Kit (Cat#Q32854, Thermo Fisher Scientific, Waltham, MA, USA). After library construction, the collected samples were sequenced on an Illumina HiSeq X platform (paired end, 150 bp).

### 2.4. Bioinformatics Pipeline

Bioinformatics pipeline analysis was performed as previously described [20]. Burrows–Wheeler Aligner (version 0.7.12) [21] was used to align the hg19 reference genome with the sequences. SAMtools was used to process the resulting alignments. The bam files were processed to distinguish somatic SNP and indel mutations using VarScan v2.4.2 [22]. ANNOVAR was used to annotate variants. Blood cell samples were used as negative controls. The copy number variation was analyzed using the CNVkit [23].

### 2.5. Statistical Analysis

Statistical analyses were conducted using SPSS software (version 19.0; IBM, Armonk, NY, USA). Heatmap and clustering analyses were conducted using Python (v3.6). Experimental data were reported as the mean ± standard error of the mean (SEM). Student's *t*-test and nonparametric tests were used for normally distributed data and nonnormally distributed data, respectively. Statistical significance was set at *P* < 0.05.

## 3. Results

### 3.1. Mutation Profile of cfDNA in Patients with CHB, LC, and HCC

**Figure 1:**
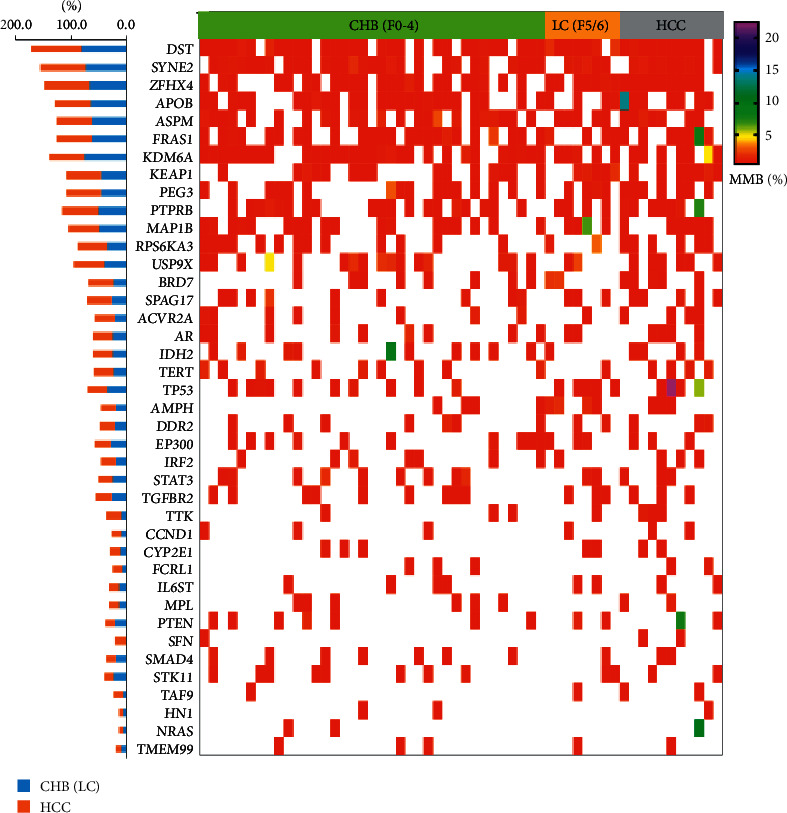
The molecular profile of plasma cfDNA among CHB, LC, and HCC patients. Significantly mutated genes were detected in plasma cfDNA of patients with CHB, LC, and HCC and were ranked in order of decreasing mutation frequency. CHB, chronic hepatitis B; LC, liver cirrhosis; HCC, hepatocellular carcinoma; and MMB: molecular mutation burden. Mutation burden of HCC-related genes increased from CHB and LC to HCC.

To investigate molecular alterations in patients with CHB, LC, and HCC, we collected blood samples from 37 patients with CHB, 8 patients with LC, and 11 patients with HCC (clinical characteristics are shown in Table 1). Plasma cfDNA and white blood cell DNA were sequenced using a targeted next-generation sequencing gene panel. The quality control is shown in Supplementary Figure 1. The mutation profile varied among patients with CHB, LC, and HCC (Figure 1). HCC-related mutations were detected during the development of CHB and LC. DST, SYNE2, ZFHX4, APOB, ASPM, FRAS1, KDM6A, and PTPRB genes were frequently mutated in patients with CHB and those with LC. DST (90.9%), SYNE2 (81.8%), ZFHX4 (81.8%), APOB (63.6%), ASPM (63.6%), FRAS1 (63.6%), KDM6A (63.6%), KEAP1 (63.6%), PEG3 (63.6%), PTPRB (63.6%), MAP1B (54.5%), RPS6KA3 (54.5%), and USP9X (54.5%) were frequently mutated in patients with HCC. These findings indicated that HCC-related gene mutations existed early in the processes of CHB and LC.

### 3.2. Mutation Burden of HCC-Related Genes Increased from CHB and LC to HCC

**Figure 2:**
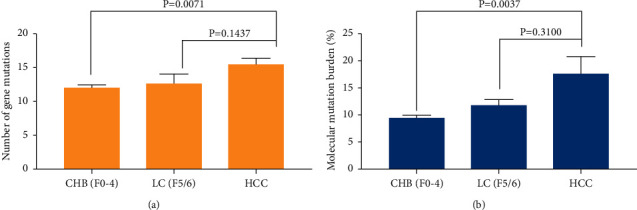
The mutated genes in patients with CHB, LC, and HCC. (a) The number of mutated genes in plasma cfDNA among CHB, LC, and HCC patients. (b) The molecular mutation burden of mutated genes among CHB, LC, and HCC patients (40 genes). CHB, chronic hepatitis B; LC, liver cirrhosis; and HCC, hepatocellular carcinoma.

To explore the gene mutation variations in the pathological progression of HCC, we analyzed the numbers and mutation burden of mutated genes in the plasma cfDNA of patients with CHB, LC, and HCC. The number of mutated genes increased from CHB and LC to HCC. The number of mutated genes in patients with HCC was significantly higher than that in patients with CHB (Figure 2(a), *P*=0.0071). There were no significant differences in the number of mutated genes between HCC and LC cases (Figure 2(a), *P*=0.1437). The mutation burden of mutated genes increased from CHB and LC to HCC. Compared with that in patients with CHB, the mutation burden was significantly increased in patients with HCC (Figure 2(b), *P*=0.0037). There were no significant differences in the mutation burden of mutated genes between patients with HCC and those with LC (Figure 2(b), *P*=0.3100).

### 3.3. Mutated Genes among Patients with CHB, LC, and HCC

**Figure 3:**
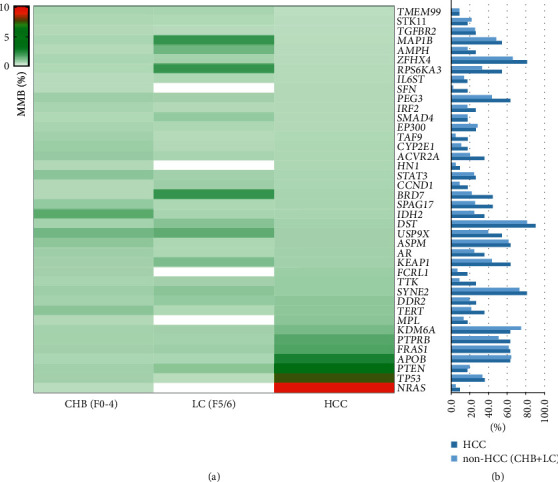
The mutation burden in cfDNA of patients with CHB, LC, and HCC. (a) The average MMB of 40 HCC-related genes among CHB, LC, and HCC patients. (b) Mutation frequency in non-HCC (CHB + LC) and HCC patients. CHB, chronic hepatitis B; LC, liver cirrhosis; HCC, hepatocellular carcinoma; and MMB: molecular mutation burden.

In patients with HCC, the average mutation burden of NRAS (10.1%), TP53 (7.4%), PTEN (4.2%), and APOB (2.6%) was the highest (Figure 3(a)). The average mutation burden of PTEN, APOB, FRAS1, KDM6A, DDR2, TTK, NRAS, TP53, PTPRB, MPL, FCRL1, HN1, and SFN gradually increased from CHB and LC to HCC (Figures 3(a) and 3(b)). The average mutation burden of NRAS, TP53, PTPRB, MPL, FCRL1, HN1, and SFN gradually increased from LC to HCC (Figure 3(a)).

**Figure 4:**
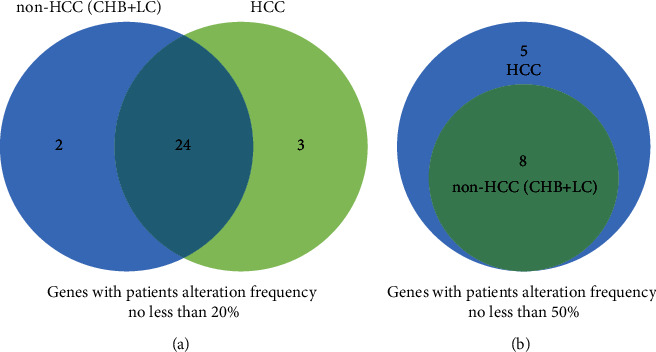
HCC-related gene mutations in HCC patients and non-HCC patients. (a) The distribution of genes with a mutation frequency >20% in HCC patients and non-HCC patients. (b) The distribution of genes with a mutation frequency >50% in HCC patients and non-HCC patients. CHB, chronic hepatitis B; LC, liver cirrhosis; HCC, hepatocellular carcinoma; and HCC, diagnosis model based on gene mutation burden.

The mutated genes varied among patients with CHB, LC, and HCC. Venn diagrams show the common and different mutated genes among patients with CHB, LC, and HCC. The mutation frequency cutoff was set at 20% (Figure 4(a)). The most commonly mutated genes among patients with CHB, LC, and HCC included DST, ZFHX4, SYNE2, PEG3, ASPM, KEAP1, KDM6A, PTPRB, FRAS1, APOB, MAP1B, RPS6KA3, USP9X, BRD7, SPAG17, ACVR2A, IDH2, AR, TERT, TP53, TGFBR2, EP300, STAT3, and DDR2. PTEN and STK11 mutations were observed in patients with CHB and LC. AMPH, IRF2, TTK, KEAP1, PEG3, MAP1B, RPS6KA3, and USP9X mutations were observed in patients with HCC (Figure 3). The mutation frequency cutoff was set at 50% (Figure 4(b)). The commonly mutated genes among patients with CHB, LC, and HCC included DST, SYNE2, ZFHX4, ASPM, PTPRB, FRAS1, APOB, and KDM6A.

### 3.4. HCC Diagnosis Model Based on Gene Mutation Burden

**Figure 5:**
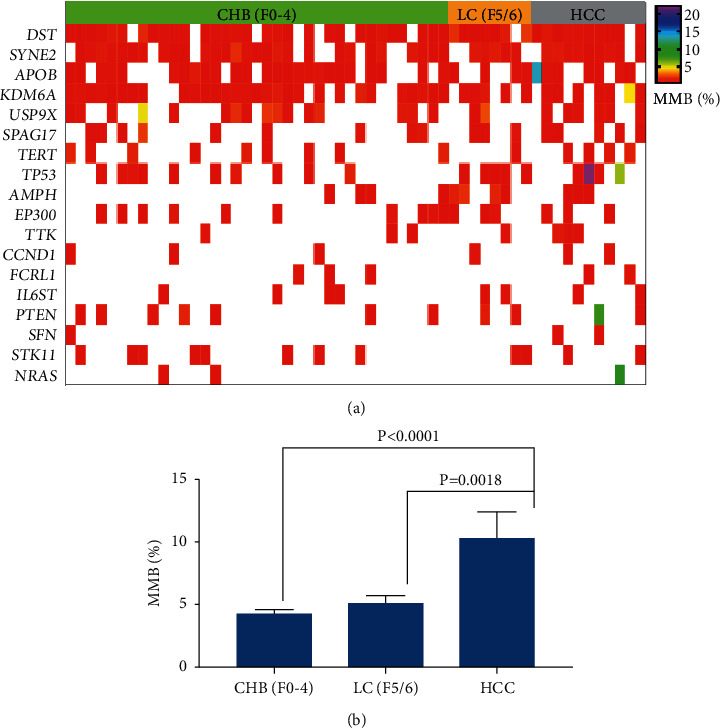
The HCC diagnosis model based on gene mutation burden. (a) The average molecular mutation burden of 18 HCC-related genes among CHB, LC, and HCC patients. (b) The molecular mutation burden of mutated genes among CHB, LC, and HCC patients (18 genes). CHB, chronic hepatitis B; LC, liver cirrhosis; HCC, hepatocellular carcinoma; and MMB: molecular mutation burden.

**Figure 6:**
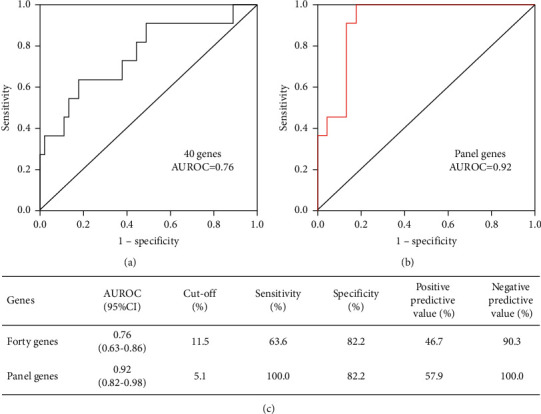
HCC diagnosis AUROC of gene mutation burden of HCC-related genes. (a) HCC diagnosis AUROC of gene mutation burden in 40 HCC-related genes. (b) HCC diagnosis AUROC of gene mutation burden in 18 HCC-related genes. (c) Diagnostic parameters of the 40-gene panel and 18-gene panel. CHB, chronic hepatitis B; LC, liver cirrhosis; HCC, hepatocellular carcinoma; and AUROC, area under the receiver operating characteristic.

The finding that the mutation burden of HCC-related genes increased from CHB and LC to HCC indicated that HCC-related gene mutations may be potential biomarkers for HCC diagnosis. Based on the gene mutation profiles among patients with CHB, LC, and HCC, we used the decision tree method to select optimal combinations of gene panels for HCC diagnosis. The optimal HCC gene panel included DST, SYNE2, APOB, KDM6A, USP9X, SPAG17, TERT, TP53, AMPH, EP300, TTK, CCND1, FCRL1, IL6ST, PTEN, SFN, STK11, and NRAS. A panel of gene mutation profiles among patients with CHB, LC, and HCC is shown in Figure 5(a). The mutation burden of panel genes in patients with HCC was significantly higher than in those with CHB and LC (Figure 5(b), *P* < 0.01 and *P*=0.0018, respectively). The gene mutation burden of 18 HCC-related genes had an area under the receiver operating characteristic (AUROC) of 0.92, with 100% sensitivity, 82.2% specificity, 57.9% positive predictive value, and 100% negative predictive value (Figure 6). The diagnostic power of the 18 HCC-related gene panels was superior to that of cfDNA (AUROC = 0.87) and alpha fetoprotein (AUROC = 0.69). The cutoff value for the HCC gene panel was 5%.

## 4. Discussion

HCC is one of the most malignant cancers worldwide and mainly occurs in East Asia and the sub-Saharan region [24]. CHB and LC are the two major risk factors for HCC. Patients with either CHB or LC could be symptomatic or asymptomatic, depending on the severity of their disease. As CHB or LC progresses, some of them will develop into HCC. Patients with HCC could obtain more clinical benefits from early therapy based on early diagnosis, reducing mortality by approximately 37% [25]. Therefore, we enrolled patients with CHB, LC, and HCC to evaluate potential biomarkers that could be employed to screen high-risk populations early.

Clinically efficient and reliable biomarkers for the early detection of HCC are critical. Alpha fetoprotein and imaging modalities are still the most frequently used means in clinics for HCC diagnosis. Nevertheless, they are not ideal for early diagnosis of HCC due to their limited specificity and sensitivity. Additionally, they lack efficiency to detect HCC at the early onset. Meanwhile, cfDNA has shown potential as a minimally invasive and efficient biomarker for risk prediction of different cancers. This study indicated that the mutation burden of HCC-related genes increased from CHB and LC to HCC.

Previous studies have indicated that liquid biopsy can be used to detect cancer by assessing circulating plasma cfDNA mutations [13, 19, 26, 27]. While most studies enrolled healthy individuals as controls, screening and continued surveillance of high-risk populations may be more meaningful in clinical practice. Early detection and confirmation of HCC in high-risk individuals usually involves different treatment strategies. Therefore, we enrolled patients with CHB, LC, and HCC. Based on the gene mutation profiles among patients with CHB, LC, and HCC, we used the decision tree method to select the optimal combination forms of the gene panel for diagnosing HCC in patients with CHB and LC. The optimal HCC gene panel included a combination of 18 HCC-related genes, which had an AUROC of 0.92, with 100.0% sensitivity, 82.2% specificity, and 100% negative predictive value, and was generally higher than the parameters in the 40-gene panel. The diagnostic power of the 18 HCC-related gene panel was superior to that of cfDNA concentrations [14] and alpha fetoprotein [28]. More importantly, the positive predictive value of this HCC gene panel for diagnosis was 57.9%, which is more powerful than in previous studies [17, 19].

Most patients with HCC have a history of chronic liver diseases, such as CHB and LC. Our study explored the plasma DNA mutation profile in the pathological progression of CHB and LC to HCC. The results indicated that HCC-related gene mutations existed as early as in CHB and LC. Furthermore, we found that the mutation burden of HCC-related genes increased from CHB and LC to HCC. In the progression of HCC, gene mutations continue to accumulate gradually. These results reveal that the plasma cfDNA mutation burden may predict disease progression in liver diseases. This finding implies that patients with CHB and LC with high molecular mutations might have a high risk of carcinogenesis.

Our study has limitations. Firstly, the sample size is relatively small. One reason for this is the difficulty to obtain a large sample size in clinics, including, but not limited to, the consent to participate in the study. Another reason for this is the purpose of the study. Our aim in this study is to explore the cfDNA mutation profile in the pathological progression of HCC and to investigate the possibility of using cfDNA in the adjunctive diagnosis of HCC. Secondly, we enrolled patients mostly at the late stage instead of early HCC such as carcinoma in situ, who are more suitable for our study. The main reason for this is the difficulty to enroll the very early patients with HCC due to the limitations of currently available diagnostic methods and the patients' late visit to clinicians. In fact, few patients with HCC are diagnosed at the early stage. We hope to solve the aforementioned limitations by enrolling more patients with CHB and LC and following their progression closely in a long time span.

## 5. Conclusions

Our study shows that the plasma cfDNA mutation burden accumulates from CHB and LC to HCC. Plasma cfDNA may contain potential biomarkers for predicting disease progression during the development of HCC. More importantly, we used the decision tree method to select optimal combinations of cfDNA mutations in the gene panel for diagnosing HCC in patients with CHB and LC. Our study broadens the knowledge of the progression of CHB and LC to HCC and deserves a prospective investigation in clinics to screen for HCC early.

## Figures and Tables

**Table 1:** Clinical characteristics of liver fibrosis and HCC patients.

Clinical characteristic	Liver fibrosis (*n* = 45)	HCC (*n* = 11)
Age, median (range)	36 (18–62)	53 (28–68)
Male, *n* (%)	31 (68.9)	10 (90.9)
cfDNA (mean ± SD, ng/ml)	16.4 ± 10.2	52.9 ± 35.4
AFP (mean ± SD, ng/ml)	12.6 ± 25	659.0 ± 1028.0
Fibrosis stages (ISHAK), *n* (%)		
F0–2	27 (60.0)	—
F3-4	10 (22.2)	
F5-6	8 (17.8)	—
Liver cirrhosis, *n* (%)	8 (17.8)	6 (54.5)

## Data Availability

Data are available on reasonable request.

## Supplementary Materials

Supplementary Materials Supplementary Figure 1. Quality control of gene-targeted NGS panel sequences. (a) The quality control score for the position of each base in the sequence fragment. The horizontal axis is the read position, the vertical axis is the average sequencing quality (Quality=−10*∗*log  10(*P*)), and *P* is the error probability. The red line is the median, the blue line is the average, and the yellow area is a 25–70% confidence interval. (b) The position distribution of the bases (A/T/G/C) in the sequencing fragment. The horizontal axis is the position of read, and the vertical axis is the percentage. (c) Size distribution of the sequencing fragment. (d) Average sequencing depth of the samples.
