# Supplementary material for: Molecular Alterations of Circulating Cell-Free DNA in the Pathological Progression of Hepatocellular Carcinoma
Source: J Oncol. 2021 Dec 3;2021:3637436. doi: 10.1155/2021/3637436 (PMC8664522; doi:10.1155/2021/3637436)

Supplementary Figure 1. Quality control of gene-targeted NGS panel sequences.

(A)The quality control score for the position of each base in the sequence fragment. The horizontal axis is the read position, the vertical axis is the average sequencing quality (Quality=-10*log10(P), and P is the error probability. The red line is the median, the blue line is the average, and the yellow area is a 25-70% confidence interval. (B) The position distribution of the bases (A/T/G/C) in the sequencing fragment. The horizontal axis is the position of read, and the vertical axis is the percentage. (C) Size distribution of the sequencing fragment. (D) Average sequencing depth of the samples.

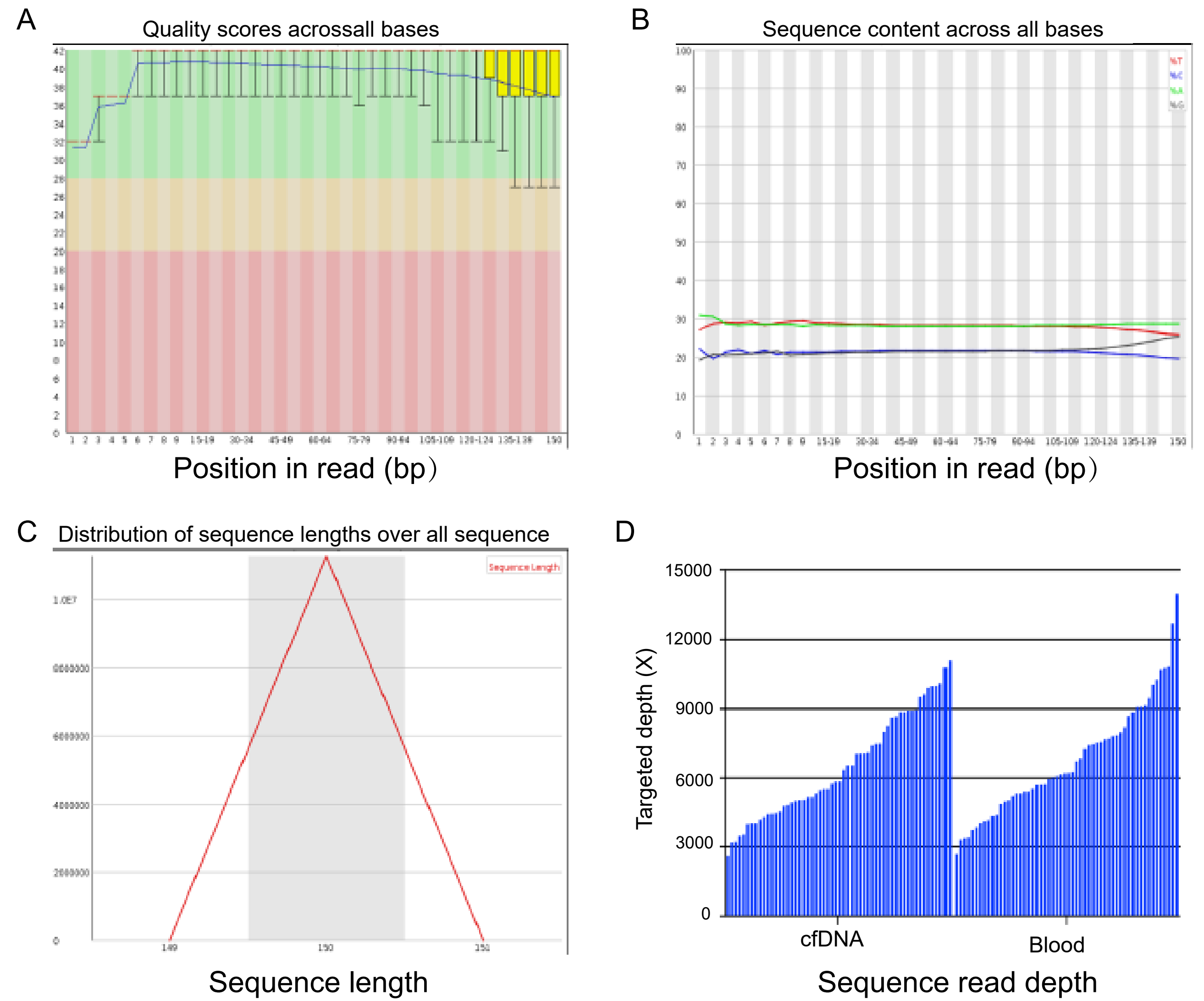

Supplement: Supplementary Materials — Supplementary Figure 1. Quality control of gene-targeted NGS panel sequences. (a) The quality control score for the position of each base in the sequence fragment. The horizontal axis is the read position, the vertical axis is the average sequencing quality (Quality=−10∗log 10(P)), and P is the error probability. The red line is the median, the blue line is the average, and the yellow area is a 25–70% confidence interval. (b) The position distribution of the bases (A/T/G/C) in the sequencing fragment. The horizontal axis is the position of read, and the vertical axis is the percentage. (c) Size distribution of the sequencing fragment. (d) Average sequencing depth of the samples. [file 3637436.f1.pdf]
